# Supplementary material for: GPR183 Is Dispensable for B1 Cell Accumulation and Function, but Affects B2 Cell Abundance, in the Omentum and Peritoneal Cavity
Source: Cells. 2022 Jan 31;11(3):494. doi: 10.3390/cells11030494 (PMC8834096; doi:10.3390/cells11030494)
Supplement: Supplementary file 1 [file cells-11-00494-s001.zip › cells-1527088-supplementary.pdf]

Supplementary figures

A

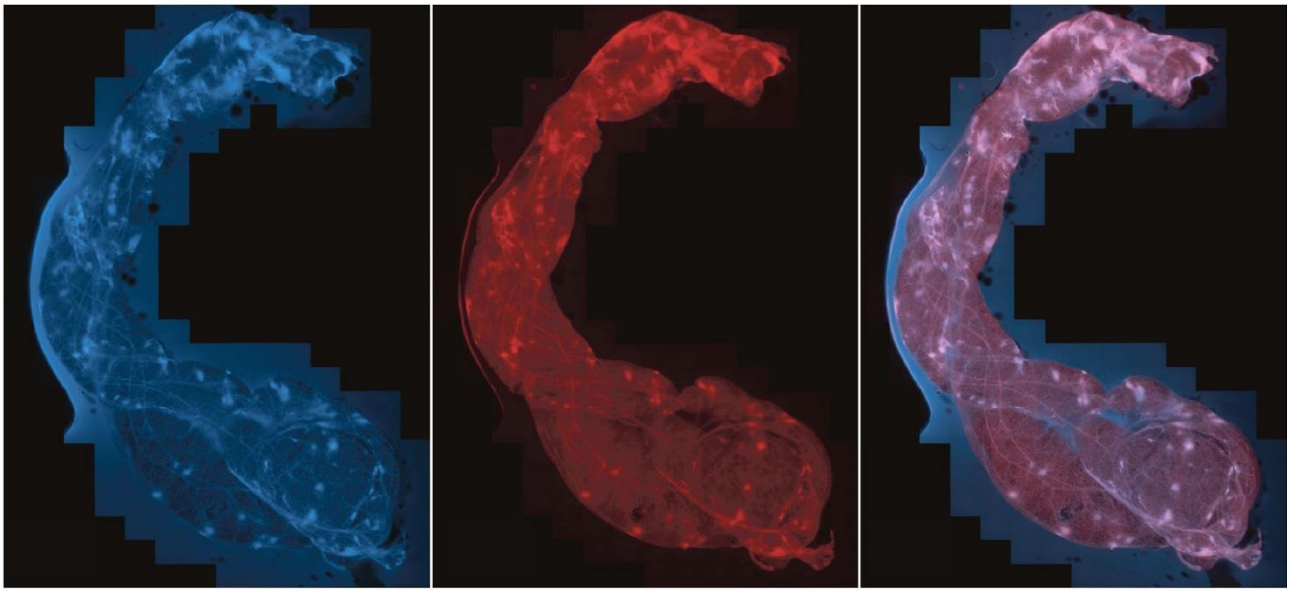

B

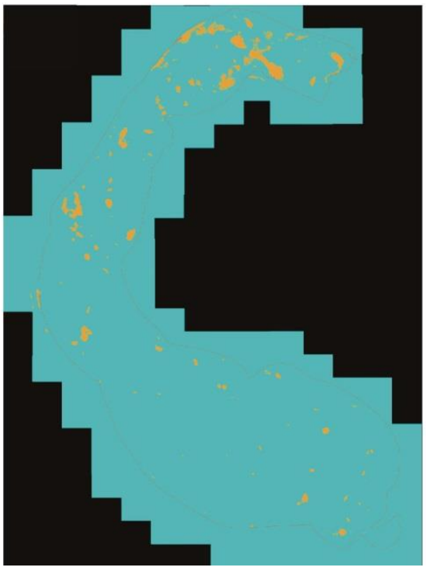

Figure S1. Omentum microscopy – supplementary figure to figure 4; (**a**) Example of an omentum stained with Hoechst (blue) and an Alexa Fluor 594-coupled anti-B220 antibody (red). The figure shows the signal obtained in channel 1 (Hoechst, left), channel 2 (B220, middle), and a combination of channels 1 and 2 (right); (**b**) Picture of 'milky spot' areas (yellow) and 'non-milky spot' areas (blue) of the omentum shown in (**a**), as predicted by the ZEN Intellisis software.

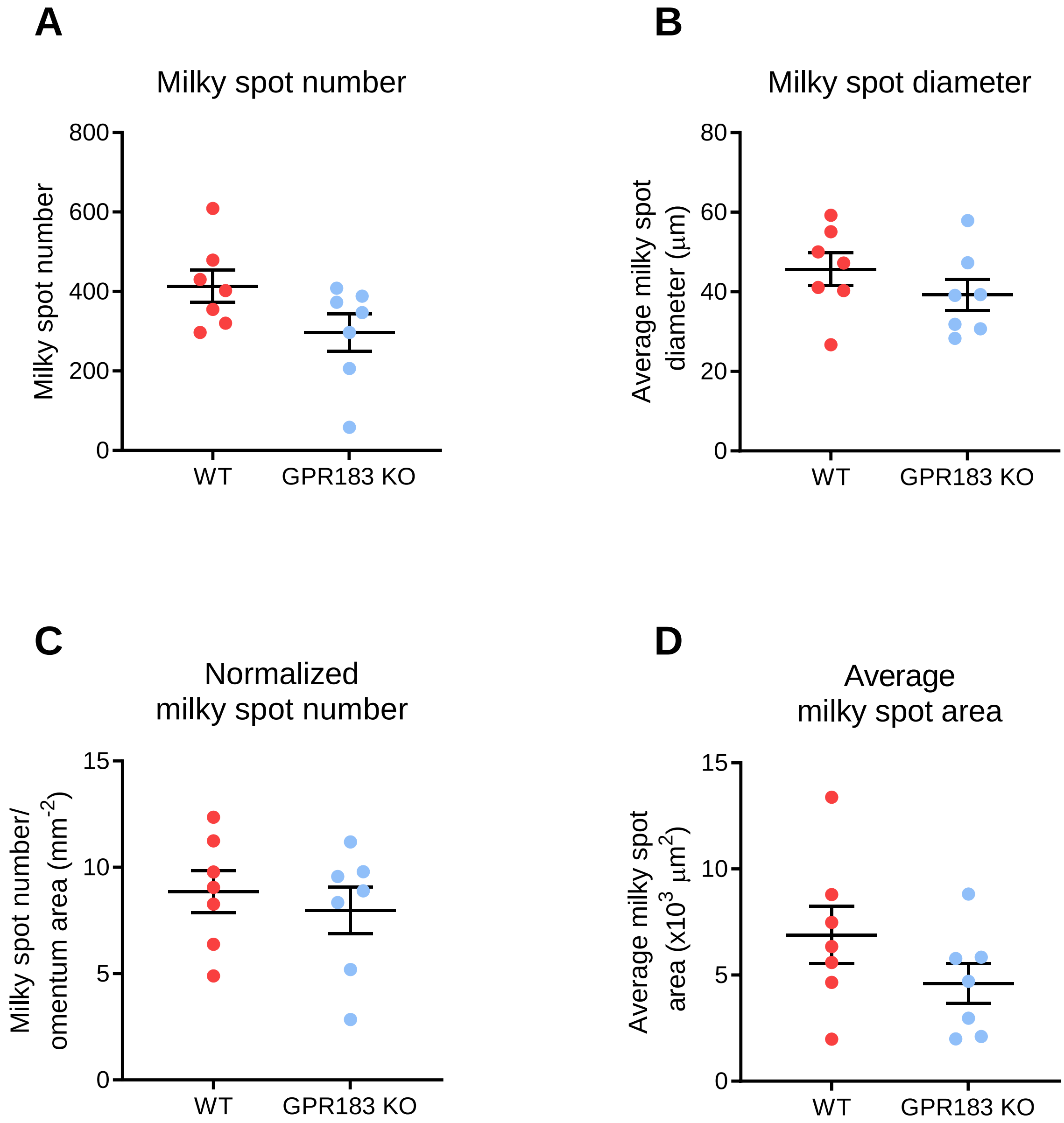

Figure S2. Omentum microscopy – second supplementary figure to figure 4. Quantification of milky spots per whole omentum from GPR183 KO and WT mice; (**a**) Total numbers of milky spots; (**b**) Average milky spot diameters; (**c**) Numbers of milky spots normalized to the omentum area; (**d**) Average milky spot area. The data were pooled from two independent experiments. Error bars represent mean ± SEM.

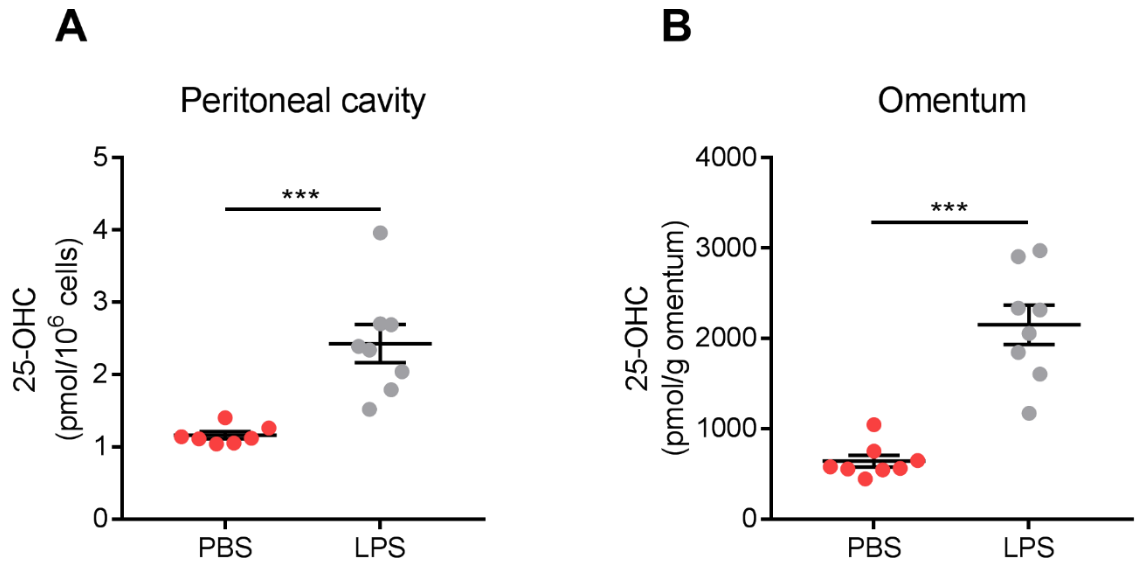

Figure S3. LPS-induced oxysterol – supplementary figure to figure 5. Mice were i.p.-injected with 10 µg LPS in PBS or PBS alone. The figure shows normalized amounts of the oxysterol 25-OHC, as measured by mass spectrometry, in peritoneal lavage; (**a**) or omentum; (**b**), 6 hours after the injections. There were 8 15 weeks old WT mice per group, but in one of the PBS-injected mice, the level of this oxysterol was below the detection limit in the peritoneal cavity, and this sample has been left out. Error bars represent mean ± SEM. ***p<0.001 by unpaired Student's t-test.

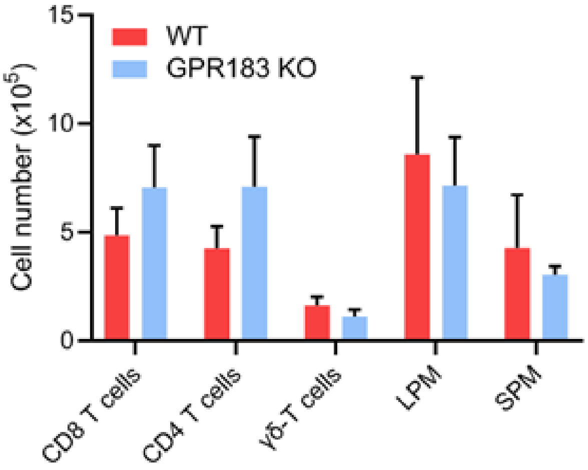

Figure S4. Peritoneal immune cell numbers after indomethacin treatment – supplementary figure to figure 6. GPR183 KO and WT mice were treated with indomethacin for a week, after which they were euthanized and their tissues analyzed. The figure shows the numbers of cells of different immune cell subsets in the peritoneal cavity, as determined by flow cytometry. LPM = large peritoneal macrophages, SPM = small peritoneal macrophages. Error bars represent mean ± SEM of data from 5-6 mice, aged 28-53 weeks.
